# Factors associated with mobile phone ownership and potential use for rabies vaccination campaigns in southern Malawi

**DOI:** 10.1186/s40249-020-00677-4

**Published:** 2020-06-05

**Authors:** Orla Marron, Gareth Thomas, Jordana L. Burdon Bailey, Dagmar Mayer, Paul O. Grossman, Frederic Lohr, Andy D. Gibson, Luke Gamble, Patrick Chikungwa, Julius Chulu, Ian G. Handel, Barend M. de C Bronsvoort, Richard J. Mellanby, Stella Mazeri

**Affiliations:** 1 Veterinary surgeon, Apt 35, The Barley House, Cork St, Dublin, 8 Ireland; 2 Mission Rabies, Cranborne, Dorset, UK; 3 Mission Rabies, Blantyre, Malawi; 4 Worldwide Veterinary Service, Blantyre, Malawi; 5 Department of Animal Health and Livestock Development, Blantyre, Malawi; 6 The Epidemiology, Economics and Risk Assessment (EERA) Group, The Roslin Institute and The Royal (Dick) School of Veterinary Studies, The University of Edinburgh, Easter Bush Veterinary Centre, Roslin, Midlothian, UK; 7 The Royal (Dick) School of Veterinary Studies, Division of Veterinary Clinical Studies, The University of Edinburgh, Hospital for Small Animals, Easter Bush Veterinary Centre, Roslin, Midlothian, UK

**Keywords:** Rabies, Mass vaccination, mHealth, Short message service

## Abstract

**Background:**

Rabies is a fatal but preventable viral disease, which causes an estimated 59 000 human deaths globally every year. The vast majority of human rabies cases are attributable to bites from infected domestic dogs and consequently control of rabies in the dog population through mass vaccination campaigns is considered the most effective method of eliminating the disease. Achieving the WHO target of 70% vaccination coverage has proven challenging in low-resource settings such as Sub Saharan Africa, and lack of public awareness about rabies vaccination campaigns is a major barrier to their success. In this study we surveyed communities in three districts in Southern Malawi to assess the extent of and socio-economic factors associated with mobile phone ownership and explore the attitudes of communities towards the use of short message service (SMS) to inform them of upcoming rabies vaccination clinics.

**Methods:**

This study was carried out between 1 October–3 December 2018 during the post-vaccination assessment of the annual dog rabies campaign in Blantyre, Zomba and Chiradzulu districts, Malawi. 1882 questionnaires were administered to households in 90 vaccination zones. The surveys gathered data on mobile phone ownership and use, and barriers to mobile phone access. A multivariable regression model was used to understand factors related to mobile phone ownership.

**Results:**

Most survey respondents owned or had use of a mobile phone, however there was evidence of an inequality of access, with higher education level, living in Blantyre district and being male positively associated with mobile phone ownership. The principal barrier to mobile phone ownership was the cost of the phone itself. Basic feature phones were most common and few owned smartphones. SMS was commonly used and the main reason for not using SMS was illiteracy. Attitudes to receiving SMS reminders about future rabies vaccination campaigns were positive.

**Conclusions:**

The study showed a majority of those surveyed have the use of a mobile phone and most mobile phone owners indicated they would like to receive SMS messages about future rabies vaccination campaigns. This study provides insight into the feasibility of distributing information about rabies vaccination campaigns using mobile phones in Malawi.

## Background

Rabies is a fatal but preventable viral disease, which causes an estimated 59 000 human deaths annually with over a third of these deaths in children less than 15 years of age [1, 2]. Domestic dogs are the cause of over 99% of human deaths from rabies and are the principal reservoir of the disease [1]. However, despite the existence of safe and effective canine vaccinations, rabies remains endemic and poorly controlled in the majority of developing countries, particularly in Sub Saharan Africa (SSA), where poor rural communities and children are disproportionately affected [1]. Consequently, control of rabies in the domestic dog population through large-scale, synchronized dog vaccination campaigns is considered the most effective approach for the elimination of human rabies [3, 4].

Rabies is endemic and inflicts a heavy burden in Malawi, causing several hundred deaths per year [2, 5, 6]. Queen Elizabeth Central Hospital in Blantyre, Malawi recorded 12 cases of pediatric rabies encephalopathy between September and November 2011, which represents double the number of cases usually seen in a year [7]. However, this likely reflects an underestimation of the true incidence of the disease as current surveillance systems in Africa are known to substantially under report rabies deaths with many cases undiagnosed or misdiagnosed [8, 9]. Furthermore, an estimated 85% of Malawians live in rural areas with limited access to health services due to geographical and socioeconomic barriers [10] and post-exposure prophylaxis treatment is often unavailable in a public health care system that is severely under resourced [7].

The control of an infectious disease through vaccination relies on vaccinating a sufficient proportion of the host population to effect ‘herd immunity’ [11, 12]. Research has shown that in order to prevent rabies outbreaks in dog populations, 40% of the population must be immune at one time. However, to maintain population immunity above this critical threshold requires a larger proportion of the dog population be vaccinated during annual vaccination campaigns [12, 13]. The World Health Organisation (WHO) recommends that to achieve control of and ultimately eliminate dog rabies, 70% vaccination coverage is required in a given area during dog vaccination campaigns [1, 4, 12, 14–17].

A major challenge to the success of rabies eradication programs is ensuring that a high proportion of the domestic dog population is vaccinated [13]. In a recent study, Mazeri et al. identified a lack of awareness about vaccination campaigns as the most commonly cited reason by dog owners for failure to attend a static point vaccination clinic [18]. Similar findings of poor attendance at vaccination clinics due to lack of public awareness have been reported in other rabies endemic countries [19–22] emphasizing the need for promotion of vaccination campaigns to the public to ensure their success.

Generating the necessary public awareness of vaccination campaigns can prove challenging in low-resource regions such as SSA, where communication infrastructure is underdeveloped. The unprecedented spread of mobile phone technologies worldwide has presented new opportunities for their use as a tool to promote health and convey healthcare information to the public [23–28]. This utilization of mobile technology in healthcare is known as ‘mobile Health’ (mHealth) [26]. According to the International Telecommunication Union (ITU) the number of mobile telephone subscriptions is already greater than the global population and almost the entire world (97%) now lives within reach of a mobile cellular signal [29, 30]. It is estimated that by 2025 the total number of mobile subscribers will reach 5.8 billion (71% of the world’s population) and the majority of new mobile subscribers will reside in developing nations [31]. In SSA, the growth rate in the mobile market is one of the highest in the world and it is anticipated that subscriber penetration rate will increase from 44% in 2018 to 50% by 2025 [31, 32]. Malawi, similar to elsewhere in SSA, has seen dramatic growth in the use of mobile phone technology over the past few years from around 16% in 2010 up to 30% mobile penetration in 2017 [33].

In the developing world, the potential for mobile phones to overcome barriers and increase access to healthcare services, especially for those in rural and underserved communities, has resulted in significant interest and investment in mHealth initiatives [23, 26, 34, 35]. In particular, the use of mobile short messaging service (SMS) to convey health information directly to individuals, is being utilized to promote public awareness about health issues such as maternal and child health, and programmes to reduce the burden of preventable diseases such as malaria and HIV/AIDS [26, 34, 36]. However, to date, the use of SMS in rabies vaccination campaigns has been limited, especially in SSA [37].

SMS demonstrates strong potential as a tool for mHealth interventions for several reasons; it is available on the most basic models of mobile phones, doesn’t require any technical knowledge or expertise to use, can be utilized in areas where there is limited electricity or internet connection, and messages can also be accessed at any time and delivered even if the phone is turned on again after a period of being switched off [36, 38–40]. Furthermore, a 2010 review found that SMS interventions for health behaviors promoted behavior change in disease prevention and management, although in this review out of nine countries represented, only one was a developing nation [38].

Previous mass rabies vaccination campaigns in Haiti have demonstrated that use of SMS vaccination alerts is an effective strategy to improve community awareness and engagement [37]. In this study we wished to examine the feasibility of using mobile phones as a means of raising public awareness of the vaccination clinics in Malawi. In order to evaluate whether or not SMS alerts could be beneficial in this setting, we surveyed communities in three districts in Southern Malawi to assess the extent of and socio-economic factors associated with mobile phone ownership, define the barriers to access of non-phone owners, and explore the attitudes of local communities towards the use of SMS to inform them of upcoming rabies vaccination clinics.

## Methods

### Ethics statement

The study has been approved by the University of Edinburgh Human Ethical Review Committee (HERC_291_18).

### Study site

Malawi is a land-locked country in south central Africa with a land area of about 118 484 km^2^ divided into three regions, north, central and south Malawi. According to the 2018 Housing and Population Census, the population of Malawi was estimated at 17.5 million, 84% of which live in rural areas [41]. The study was conducted in three adjacent districts within southern Malawi; Zomba, Blantyre and Chiradzulu. The economy in these districts is dominated by agriculture, with the rural regions being divided in small landholdings. Blantyre district has a human population of 1 251 484 inhabitants, of which nearly 64% live in the urban area [41]. Zomba district has 851 737 inhabitants, around 12% of which live in the urban area [41]. Chiradzulu is a mainly rural district with a population of 356 875 inhabitants [41]. Blantyre city and Zomba city are the second and fourth biggest cities in the country, respectively [41].

According to the Human Development Index, Malawi is one of the poorest nations in the world, ranking 172 out of 189 countries [42]. With a human development index of 0.485 the country is classified as a low human development country [42]. Of the population, 52% live below the national poverty line and 29% are considered “ultra-poor” [42]. There are regional variations in poverty rates, with the Southern region of Malawi poorer than the North or Central region. Poverty is also worse in rural compared to urban areas, with about 57% of the rural population living in poverty compared to 17% of the population in urban areas [43].

The overall literacy rate of the population aged 15 years and older is 65% [43], however literacy rates vary between the three study districts; Chiradzulu (76%), Blantyre (72%), Zomba (69%), and also between rural and urban areas, with comparatively higher literacy rates seen in urban areas than rural areas (87% in Zomba city, 92% in Blantyre city) [43].

There are two main mobile phone operators in Malawi, Airtel Malawi Limited and Telekom Networks Malawi Limited (TNM), and mobile phone signal coverage is reported to reach over 80% of the country [44]. All SIM cards in Malawi need to be registered on a central database, and a customer’s national identity number needs to be verified when purchasing, replacing, or swapping a SIM card [33].

This study was carried out in parallel with the Mission Rabies vaccination campaign, which is described elsewhere [6, 18, 45]. Briefly, since 2015, Mission Rabies organises annual mass dog rabies vaccination campaigns covering the three districts, which were chosen due to the high number of paediatric rabies cases reported there [7]. At the end of each campaign, in order to assess vaccination coverage achieved, post-vaccination household surveys are conducted. In this paper we describe data collected during the 2018 campaign, where surveys were carried out between 1 October–3 December 2018. For the 2018 campaign, the study area was split into 576 ‘vaccination working zones’ and 90 of these zones were randomly sampled in order to carry out post-vaccination surveys. This sample size was chosen based on the methodology used for the rabies vaccination campaign whereby the aim is to randomly sample at least 10% of the vaccination zones of each district and city each year. Sampling was stratified by district. Data collectors visited one out of every four houses in each working zone, counting houses on either side of the road. If a house was unavailable for any reason they moved to the next house. In order to assess the potential of using mobile phones to inform people about the campaign, 2018 surveys included two components, the first related to dog vaccinations status and the second related to mobile phone ownership and use.

### Data collection

During the post-vaccination surveys, questionnaire data were collected using the Worldwide Veterinary Service (WVS) Smartphone App [46]. Regular post-vaccination surveys enquire information related to the number of people in the household, the number of dogs owned and the vaccination status of those dogs. For the purposes of the current study, a questionnaire related to mobile phone ownership was appended to the regular post-vaccination surveys, which is available as Additional file 1. Briefly, this questionnaire included questions related to the respondent and the household such as age, gender, education level, religion, number of residents and whether they owned or had access to a mobile phone. “Access” was defined as “use of a mobile phone that is owned by another individual”. Additional information was asked about the type of mobile phone, frequency and purpose of use and reasons for not owning a mobile phone. Finally, respondents were asked how they find out about events in the local community.

### Other data sources

Poverty data were sourced from two WorldPop raster datasets “mwi11povcons125.tif” and “mwi11povcons200.tif” [47], where 2010–2011 estimates of proportion of people per grid square living in poverty, as defined by US dollars 1.25 a day and US dollars 2 a day thresholds respectively, are available. Population density data were sourced from the Malawi Spatial Data Platform (MASDAP) Population Density of Malawi raster dataset [48].

### Data analysis

All data analysis was carried out within the R statistical software (www.r-project.org) environment version 3.6.1 [49]. Specific packages used are mentioned below.

### GIS data extraction

GPS coordinates recorded for each household were used to extract the GIS data for that dog. The package *sp* [50] was used to extract data from shapefiles, while package *raster* [51] was used to extract data from raster files.

### Multivariable logistic regression model

A multivariable logistic regression model was built using mobile phone ownership as the dependent variable. Explanatory variables included respondent’s gender, age, education, population density, proportion of population living in poverty and district.

The dataset was split into a training dataset (70%), which was used to build the model and a test dataset (30%), which was used to validate the model using the *caret* package [52]. Since only a small number of explanatory variables were available, all of them were considered for the final model, using the dredge function from *MuMIn* [53] package, which provides all possible variable combinations. Model selection was based on lowest Akaike information criterion (AIC). Five-fold cross-validation was used to confirm the final model selected based on the area under the curve (AUC) using package *vtreat* [54]. The final model was validated, testing its ability to predict phone ownership in the test dataset by estimating the area under the curve using package *ROCR* [55].

## Results

### Demographics

A total of 1882 post-vaccination questionnaires were completed across Blantyre (497), Zomba (958) and Chiradzulu (427) districts. Of the respondents, 860 (46%) were male and 1018 (54%) were female. Four respondents did not state their gender. The median age of respondents was 35 years, which ranged from 18 years to 89 years.

### Phone ownership

**Fig. 1:**
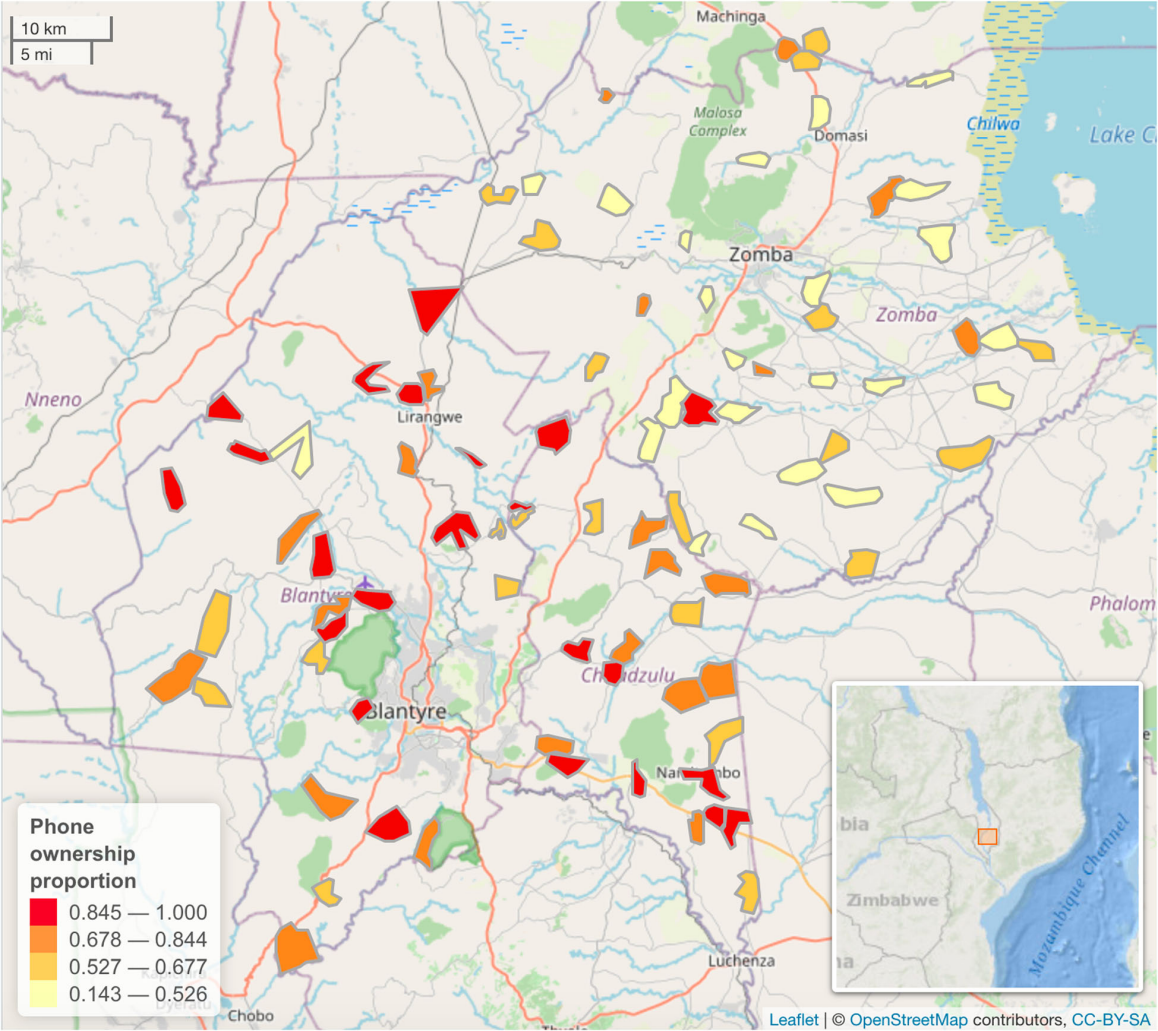
Geographical location of study areas and phone ownership patterns. Map shows phone ownership proportion in each working zone surveyed. ﻿The map was plotted using R package leaflet [56] using tiles sourced OpenStreetMap

Out of all respondents, 36 individuals chose not to say if they owned a mobile phone (2%). Out of the 1846 who replied, 1093 (59%) owned a mobile phone, 134 (7%) had access to a mobile phone and 618 (33%) did not own or have access to a mobile phone. By age, the percentage of mobile phone ownership was highest among the 26–40 years age group, 69.7% of which owned a mobile phone, followed by the 41–55 years group (67.3%), the 18–26 years age group (56.4%), and was lowest in the > 55 years age group (48.4%) (Additional file 2). Looking at mobile phone ownership by district; in Blantyre 76% owned a mobile phone and 6% had access to a mobile phone, in Chiradzulu district 62% owned a mobile phone and 20% had access, and in Zomba 49% owned a mobile phone and 2% had mobile phone access. Figure 1 shows a map of the location of zones surveyed as well as the mobile phone ownership patterns observed in those. Mobile phone ownership varied with education level with 85% of more educated people (secondary education or more) owning mobile phones compared to 61% of people with less than secondary education (Additional file 3).

### Mobile phone sharing

Mobile phone owners were asked whether they shared their phone with anyone else. Most respondents who owned a mobile phone did not share phone access with anyone else (58%). For mobile phone owners that did share access to their phone with other users, the number of users shared with ranged from one up to 30 users, with a median of three users.

### Phone usage

Questionnaire respondents were also asked to provide information on the frequency of their mobile phone usage. Most mobile phone owners (91%) said they use their phone every day, 3% every other day, 5% once or twice per week and less than 1% said they use their phone less often. Among those who had access to someone else’s phone, 77% said they use it every day, 8% every other day and 14% once or twice per week. Mobile phone owners were also asked to indicate the time of day of phone usage and 73% said they use it all day. Of those who did not use their phones all day, afternoons and evenings were more popular (Additional file 4).

### Phone type, SMS and WhatsApp use

Questionnaires provided information regarding the type of mobile phone, basic model or smart phone that respondents owned or had access to. It was found that most mobile phone owners had a simple feature, basic model phone (84%) and that few owned a smart phone (15%). Of those who did not own their own mobile phone but had access to one, most had access to a basic model phone (87%) and only 7% had access to a smart phone. A small percentage did not know which type of mobile phone they owned or had access to. The questionnaire also looked at WhatsApp and SMS use among mobile phone users. It was found that 92% of those who either owned or had access to a mobile phone used SMS as a form of communication, while only 15% used WhatsApp. The reasons given for not using SMS are shown in Additional file 5. The most common reason provided was illiteracy. Other reasons included digital illiteracy, that they did not like using SMS, visual impairment and that it takes too long to respond.

### Barriers to mobile phone ownership

The 618 respondents who did not own a mobile phone were asked reasons why (Additional file 6). The principal reason identified for not owning a mobile phone was the cost of the phone itself, mentioned by 81% of respondents. Other reasons given included; not useful 11%, digital illiteracy 9%, poor mobile phone network signal 4%, cost of 3G data 0.49% and illiteracy 0.32%.

### Means of information communication in local communities and attitudes toward SMS text alert

**Fig. 2:**
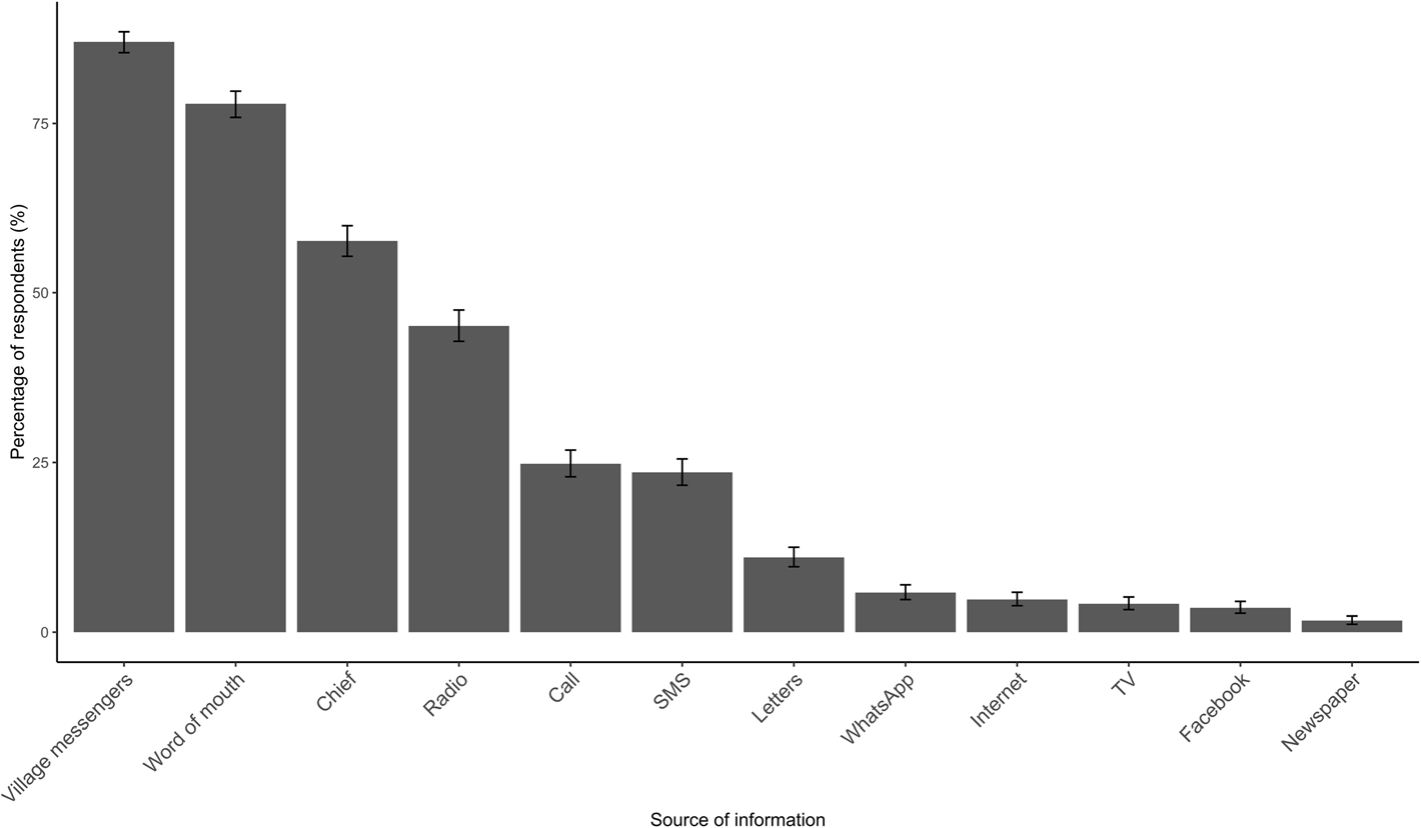
Sources of information about events in the local community. Respondents could choose more than one option

Respondents were asked about how they find out about events in the local community and more than one response was allowed (Fig. 2). Respondents indicated that more traditional, ‘low tech’ means of communication such as village messengers (87%), word of mouth (78%) and the village chief (58%) were the predominant means of information sharing. ‘Village messengers’ are defined as a person whose responsibility is the distribution of information from the chief (traditional authority) to the public (within the area of the chief’s control) and is paid by the chief’s office. Less frequent but still common means of information spread were via technologies including radio (45%), phone call (25%) and SMS (24%). Other methods of communicating information less commonly identified by respondents were post/letters, WhatsApp, internet, TV media, Facebook and print media (newspaper). Attitudes towards future text reminders about rabies vaccination campaign were also surveyed. A majority of respondents who owned or had access to a mobile phone indicated that they would like to receive SMS reminders about rabies vaccination campaigns (99.6%).

### Multivariable logistic regression model

**Fig. 3:**
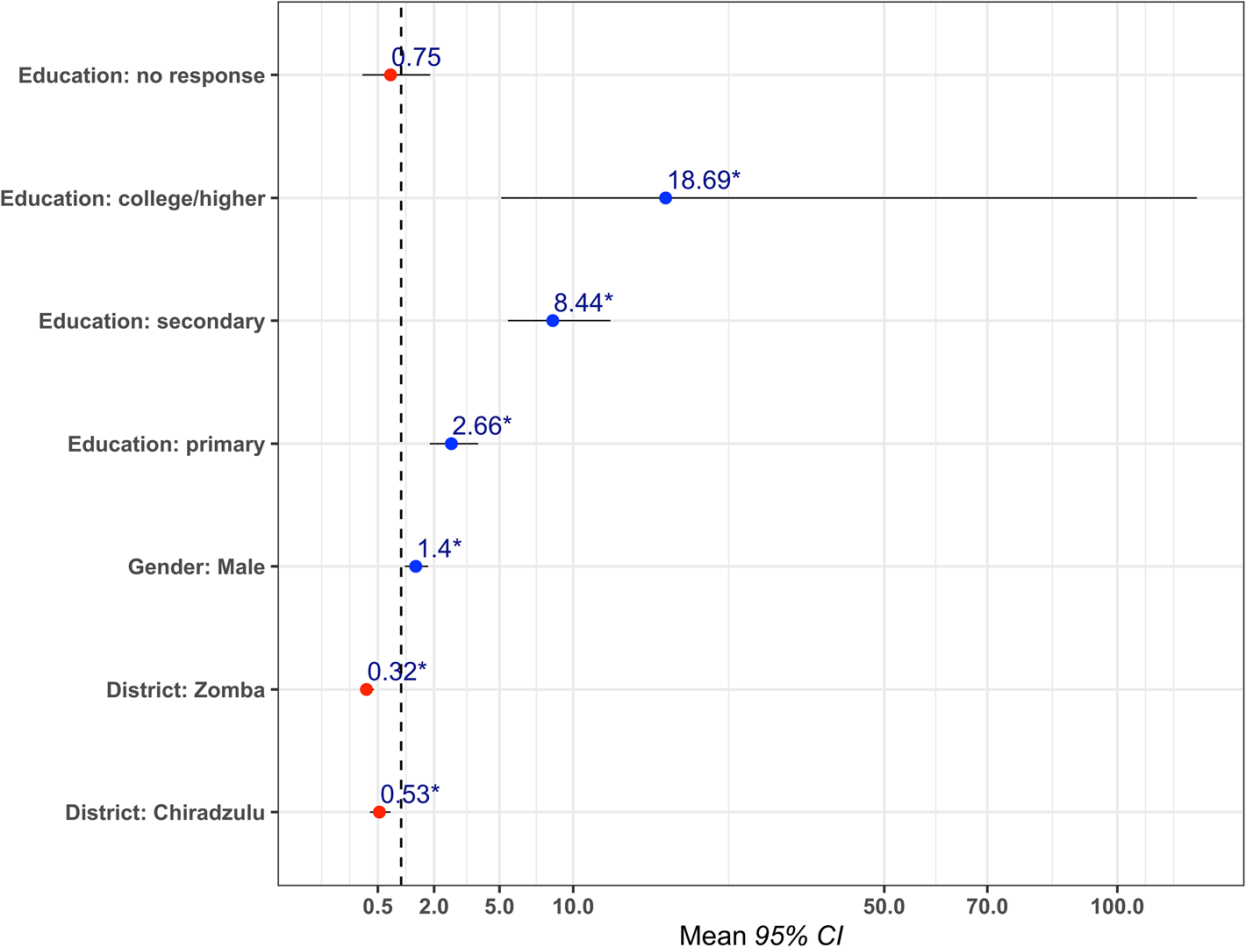
Multivariable logistic regression model predicting phone ownership. Figure shows estimates of the odds ratios (dot) and 95% confidence intervals (lines) for each variable. * corresponds to a *P* value < 0

The results of the final multivariable logistic regression model predicting mobile phone ownership are shown in Fig. 3. Numerical results of the regression model are shown in Additional file 7. The model shows that the odds of owning a mobile phone increased with higher education levels, compared to those with no education. Compared to those residing in Blantyre district, those residing in Zomba or Chiradzulu districts were less likely to own a mobile phone. Lastly, being male was positively associated with mobile phone ownership. The model has an area under the curve (AUC) of 0.69, indicating moderate predictive power.

## Discussion

In our study we assessed respondent’s attitudes towards an SMS vaccination reminder campaign. The response was overwhelmingly positive towards SMS reminders and a large majority of mobile phone users indicated that they would like to receive SMS reminders about future rabies vaccination campaigns. Previous studies using SMS messaging reminders for vaccination found a similarly positive response among participants [37, 57]. A review of mHealth projects in Africa found the principal reason given for highly positive perception of mHealth projects was a high acceptance and familiarity with the use of mobile phones [23].

In our study, two thirds of those surveyed stated they either owned or had access to a mobile phone, with basic feature phones by far the most common type of mobile phone in use. This is higher than was reported in a recent countrywide census, which estimated the total households in Malawi with a mobile phone at around 52% [41]. However, like elsewhere in SSA, mobile phone ownership in Malawi does not equate with the number of mobile phone users as phone sharing is commonplace and allows non-mobile phone owners to have access [24]. Previous surveys have estimated mobile phone subscriber penetration rate at 30% [33], but comparison with these surveys is problematic since estimated subscriber rates are based on the number of SIM cards registered and mobile phone owners may possess more than one SIM card. Additionally, non-mobile phone owners may have a SIM card that they use on another person’s phone. In our study, respondents were asked about mobile phone ownership and access but not about SIM card ownership. As such, it is unclear how frequent it is for individuals to have multiple SIM cards and what impact this would have on any mHealth campaign.

The vast majority of mobile phone users were able to use SMS with much fewer using WhatsApp, reflecting the fact that few respondents had smartphones with 3G capabilities. These findings are consistent with mobile data from other SSA nations, where most of the population own mobiles, but smartphone adoption is more modest [31, 58]. Consequently, SMS has the potential to reach a much larger proportion of the community than a 3G-based messaging services such as WhatsApp. The results of our study provide evidence of the widespread use of SMS technology among mobile phone users in the three districts surveyed in Malawi, nonetheless, there were a considerable proportion who did not have access to a mobile phone, and this represents a major limitation for the use of SMS vaccination alert campaign in Malawi.

Despite unprecedented growth in mobile phone ownership across SSA, a digital divide persists, with lower levels of phone ownership among individuals from the most marginalised groups [58, 59]. Our results show similar trends to those seen in other SSA countries where educational, geographical, generational and gender gaps in mobile phone ownership exist [53]. In our study, a higher education level, was associated with greater levels of mobile phone ownership. A 2015 report by Malawi Communication Regulatory Authority (MACRA) on information and communication technology (ICT) in Malawi found that while 74% of individuals with a tertiary education or higher had access to an internet enabled mobile phone, only 20% of those with primary education had access to such a device [60].

We found that mobile phone ownership was highest among the 26–40 year age group. We suspect this is because this age group are old enough to be able to afford a mobile phone but young enough to embrace new technologies. As is the case in many developing nations, the population in Malawi is young, with an average life expectancy of 63.8 years [42] and children under age 15 represent 48% of the household population, while individuals age 65 and older represent only 4% [61].

Gender was also found to be a factor in mobile phone ownership with males more likely to own mobile phones than females. Gender gaps in mobile ownership are reflective of existing gender inequalities and a recent report indicated that women in SSA are 15% less likely to own a mobile phone than men [59].

In most of SSA, rural areas tend to have lower mobile penetration than urban areas and the rural gender gap is also wider [31]. Geographical differences in phone ownership have previously been reported in Malawi, with fewer mobile phone owners in the more densely populated southern and central regions compared to the northern region. Previous surveys have also identified a marked divide in mobile phone ownership in Malawi between urban and rural households [62].

In our study geographical location had an impact on mobile phone ownership, with those residing in Zomba or Chiradzulu less likely to own a phone than those living in Blantyre. The large difference in phone ownership in Blantyre and Zomba districts is hypothesised to be related to differences in socico-economic status in the population between these two districts. Severe poverty is widespread in Zomba district and around 70% of the population live below the national poverty line, making it one of the poorest districts in Malawi [63]. Wealth has been found to be a factor in the rate of mobile phone ownership elsewhere in SSA, with higher income individuals more likely to own a mobile phone than those with lower incomes [58]. There is also a lower literacy rate in Zomba compared to Blantyre and Chiradzulu [43]. Our study found that both these factors have an impact on mobile phone ownership, with phone cost and illiteracy two of the principal barriers cited by respondents as reasons for not owning a mobile phone. Additionally, almost 64% of the population of Blantyre district live in the urban area compared to only 12% of population of Zomba district [41]. This could also potentially influence mobile phone ownership as those living in urban areas have been shown to have higher literacy levels, greater access to electricity and lower levels of poverty [43]. Overall only 11% of Malawian households use electricity as their main energy source in the home [41], and in rural areas this figure drops to just 3% of households compared to 33% of urban households [43]. This presents a challenge to mobile phone users, especially in rural areas, who may struggle to charge a mobile phone. Low disposable household income, rural location and limited access to electricity are all factors likely to play a part in the lower levels of phone ownership found in the Zomba region.

Knobel et al. reported socio-economic factors associated with dog ownership in Tanzania and found that households that were better educated, wealthier and larger were more likely to own dogs [64]. Other studies have reported lower rates of dog ownership in impoverished rural areas [9, 45, 65]. The implication of this being that those households that are more likely to own a mobile phone are more likely to own a dog and therefore benefit from an SMS vaccination alert. However, in this study we did not analyse how factors such as poverty and education level impact on dog ownership in the three districts surveyed and so are unable to draw conclusions on whether those owning mobile phones are more or less likely to also be dog owners.

Cost of a mobile handset was overwhelmingly identified as the single most important barrier to mobile phone ownership in this study, indicating that poverty is a significant obstacle to mobile phone ownership in the communities surveyed. Affordability of phones is commonly cited as the principle barrier to mobile ownership across many low and middle-income countries [59] and women tend to report handset and credit cost as a barrier more often than men [66]. Apart from cost-related barriers, issues with illiteracy, digital literacy and poor phone network signal were also cited as reasons for not owning a mobile phone. In most countries, the main barriers to mobile ownership after cost tend to be difficulties with reading and writing and using mobile handsets [59]. Previous studies in Malawi have similarly reported that a lack of disposable income and lack of digital literacy are key barriers to mobile ownership, especially among women [33].

We found that more traditional, low-tech means of communications such as ‘word of mouth’, village messengers and radio are still the predominant method of information spread in the communities surveyed. Despite growth in the use of the internet and mobile phones, radio is still the most widely used medium for news in SSA [67, 68] and has the advantage of being able to reach those with poor literacy or low disposable income. Furthermore, radio can be broadcast in the local language and radio listening is often a shared experience, which increases the chances for information to be disseminated. The 2015 MARCA ICT report found that 96% of Malawians listen to the radio and those with little or no education tend to listen to radio more frequently [60]. Although mobile phone use is becoming more ubiquitous in Malawi, a significant proportion of individuals are reliant on more traditional channels for information spread and the importance of these should not be overlooked. This is not to say that an SMS vaccination reminder campaign would not be effective, however, it may be necessary to integrate other communication channels alongside any mobile phone campaign. An SMS vaccination alert campaign in Haiti found that, although text messages were the most frequently cited method of promoting awareness among participants, megaphones and word of mouth were also important methods [37].

We are mindful of the limitations of our survey approach, which was confined to three districts in the Southern region of Malawi. Comparing the Southern region to the rest of Malawi, there is more poverty in the districts of the Southern region than there are in the Northern and Central regions [43]. Additionally, both Blantyre and Zomba district have cities, which is likely to impact on the ability to extrapolate the results to other districts.

## Conclusions

A lack of public awareness about rabies vaccination campaigns has been shown to be a key barrier to vaccination attendance by dog owners in Malawi. This study looks at mobile phone ownership and usage in three districts in southern Malawi and surveys the attitudes of mobile phone users to an SMS vaccination reminder campaign. The findings indicate that a majority of people have the use of a mobile phone in the districts surveyed, however, similar to other SSA countries, there is evidence of a mobile phone divide, where those with higher socioeconomic status have greater access. The overall attitude of those surveyed towards receiving SMS reminders was positive and a majority of phone owners indicated they would like to receive SMS messages about future rabies vaccination campaigns. However, our findings also show that despite massive growth in the use of mobile phones among developing nations, it is premature to assume widespread access to mobile phones in impoverished rural regions. We suggest that although SMS reminders have the potential to increase public awareness of rabies vaccination campaigns, we should not ignore more traditional channels for informing communities. And in order to successfully reach those with lower socioeconomic status it is vital to integrate other forms of communication beyond mobile phones, such as radio, village messengers and village chiefs. In summary, this study provides an important insight into the potential feasibility of distributing information about rabies vaccination campaigns using mobile phones in Malawi.

## Supplementary information

**Additional file 1.** Post-vaccination survey with questions specific to phone ownership study.

**Additional file 2.** Figure showing the distribution of respondent’s age in years.

**Additional file 3.** Figure showing phone ownership according to education status.

**Additional file 4.** Figure showing phone usage according to time of day.

**Additional file 5.** Figure showing reasons respondents did not use SMS.

**Additional file 6.** Figure showing reasons given for not owning a mobile phone.

**Additional file 7.** Table showing multivariable logistic regression model predicting phone ownership.

## Data Availability

Relevant data are available on request. Household GPS locations will be removed from the datasets as could be considered personally identifiable information, but data extracted using GPS locations have been provided.

## Abbreviations

SSA: Sub Saharan Africa
SMS: Short message service
WHO: World Health Organisation
mHealth: mobile Health
MACRA: Malawi Communication Regulatory Authority
ICT: Information and communications technology
