# Supplementary material for: Factors associated with mobile phone ownership and potential use for rabies vaccination campaigns in southern Malawi
Source: Infect Dis Poverty. 2020 Jun 5;9:62. doi: 10.1186/s40249-020-00677-4 (PMC7275584; doi:10.1186/s40249-020-00677-4)
Supplement: Supplementary file 1 — Additional file 1. Post-vaccination survey with questions specific to phone ownership study. [file 40249_2020_677_MOESM1_ESM.docx]

**Additional File 1: Post-vaccination survey with questions relating to mobile phone ownership**

Phone Access

1. Gender of respondent
2. Age of respondent
3. What is the highest level of education you have achieved:
   1. No Education
   2. Some Primary
   3. Complete Primary
   4. Some Secondary
   5. Complete Secondary
   6. College
   7. Higher
   8. No_Response
   9. N/A
4. What is the highest level of education achieved by any member of your household:
   1. No Education
   2. Some Primary
   3. Complete Primary
   4. Some Secondary
   5. Complete Secondary
   6. College
   7. Higher
   8. No_Response
   9. N/A
5. Do you own or have access to a mobile phone?
   1. Own
   2. Access
   3. No
   4. Prefer not to say
6. If own/access what type of mobile phone?
   1. Simple/Feature phone
   2. Smart phone
   3. Don’t know
7. If own, does anyone else have access to this phone?

A. Yes (number of people)

B. No

1. How often do you use your phone?
   1. every day
   2. every other day
   3. 1-2 times per week
   4. less often
2. Which times of the day do you use your phone?
   1. early morning
   2. mid-morning
   3. lunchtime
   4. afternoon
   5. evenings
   6. All day
3. Do you ever use SMS as a form of communication?
   1. Yes
   2. No
4. If not, why not? (free text)
5. Do you ever use WhatsAPP as a form of communication?
   1. Yes
   2. No
6. Why don’t you have a phone (click all that apply?)
   1. It’s not useful
   2. Cannot afford a phone
   3. Cannot afford airtime
   4. Cannort afford data
   5. Don’t know how to use a phone
   6. Poor signal
   7. Other
7. How do you find out about events in the local community (tick all that apply)?
   1. Radio
   2. In person (Word of mouth)
   3. Telephone call
   4. SMS
   5. Internet
   6. Village messengers
   7. Letters
   8. Chief
   9. WhatsAPP
   10. Facebook
   11. Newspaper
   12. Television
8. Would you like to receive text message reminders about rabies vaccination campaigns in the future?
