# Supplementary figures and images for: Factors associated with mobile phone ownership and potential use for rabies vaccination campaigns in southern Malawi
Source: Infect Dis Poverty. 2020 Jun 5;9:62. doi: 10.1186/s40249-020-00677-4 (PMC7275584; doi:10.1186/s40249-020-00677-4)

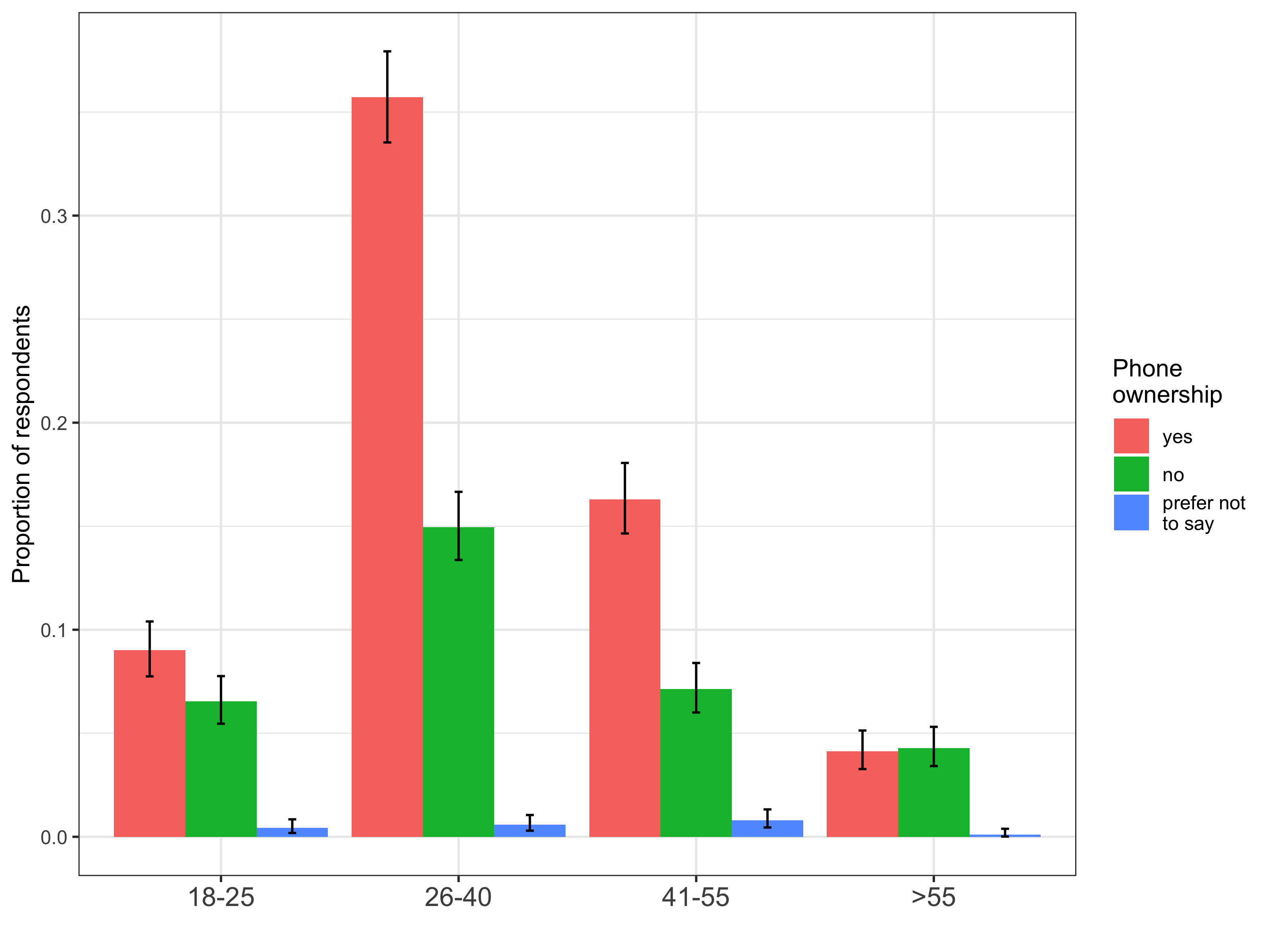

Supplement: Supplementary file 2 — Additional file 2. Figure showing the distribution of respondent’s age in years. [file 40249_2020_677_MOESM2_ESM.png]

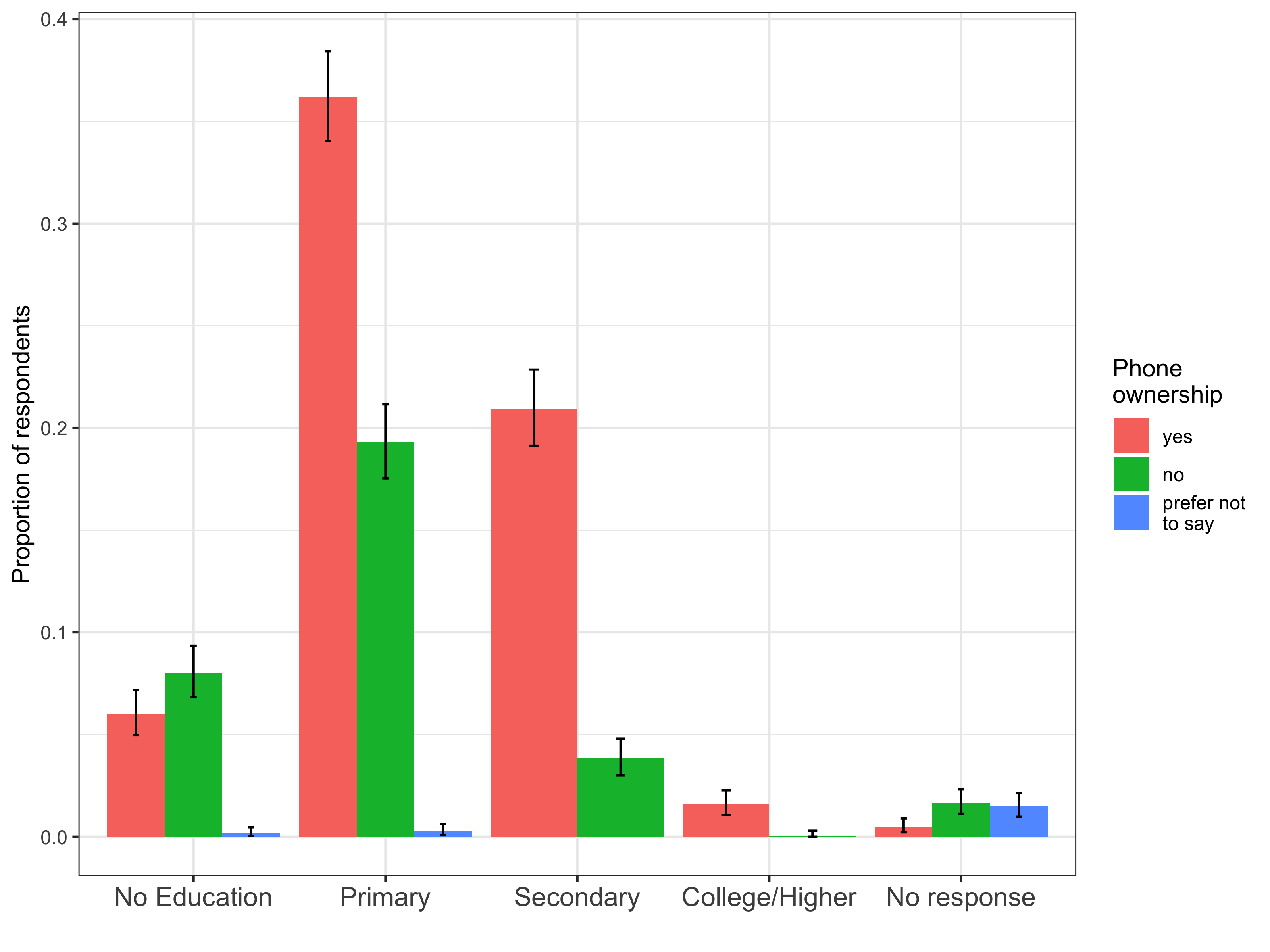

Supplement: Supplementary file 3 — Additional file 3. Figure showing phone ownership according to education status. [file 40249_2020_677_MOESM3_ESM.png]

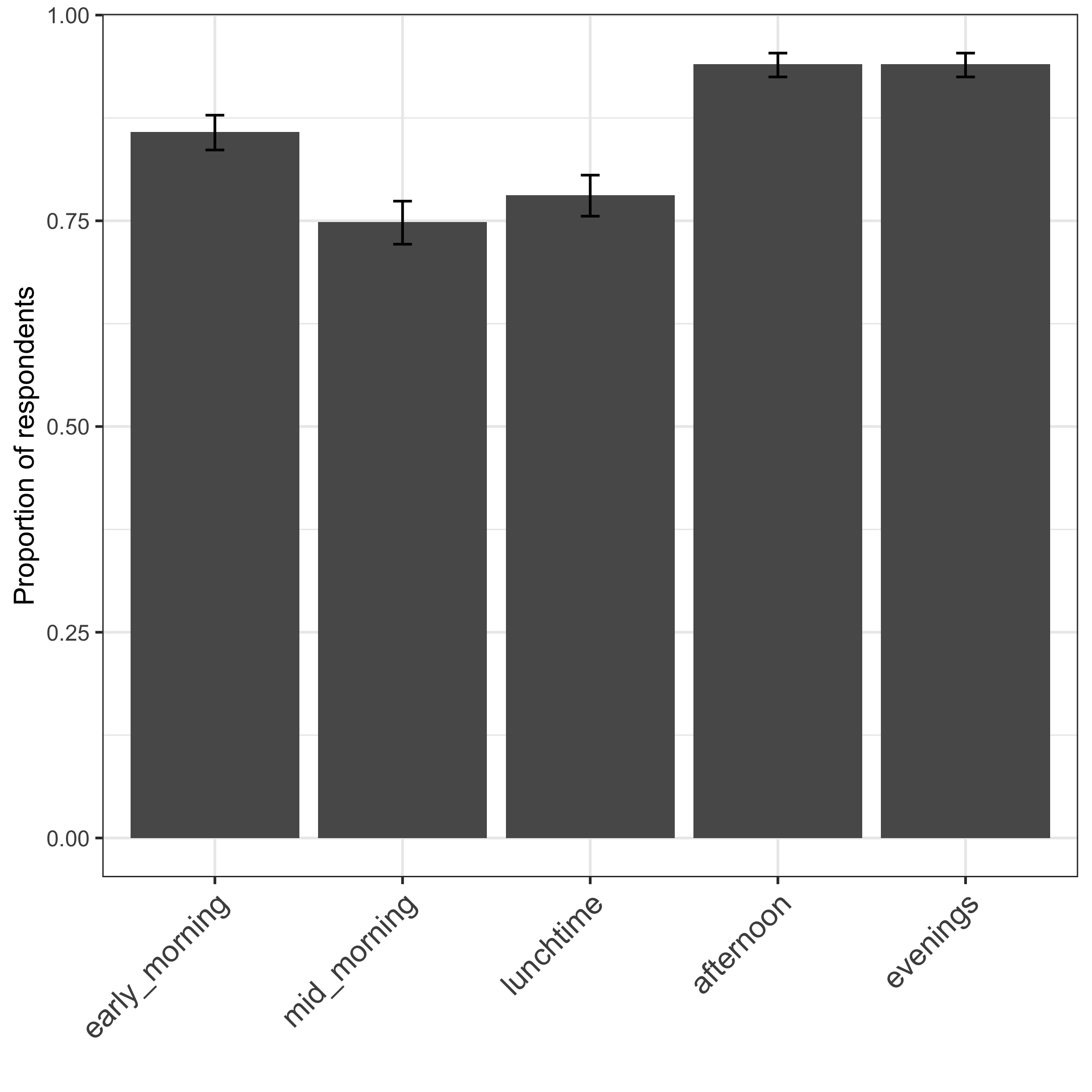

Supplement: Supplementary file 4 — Additional file 4. Figure showing phone usage according to time of day. [file 40249_2020_677_MOESM4_ESM.png]

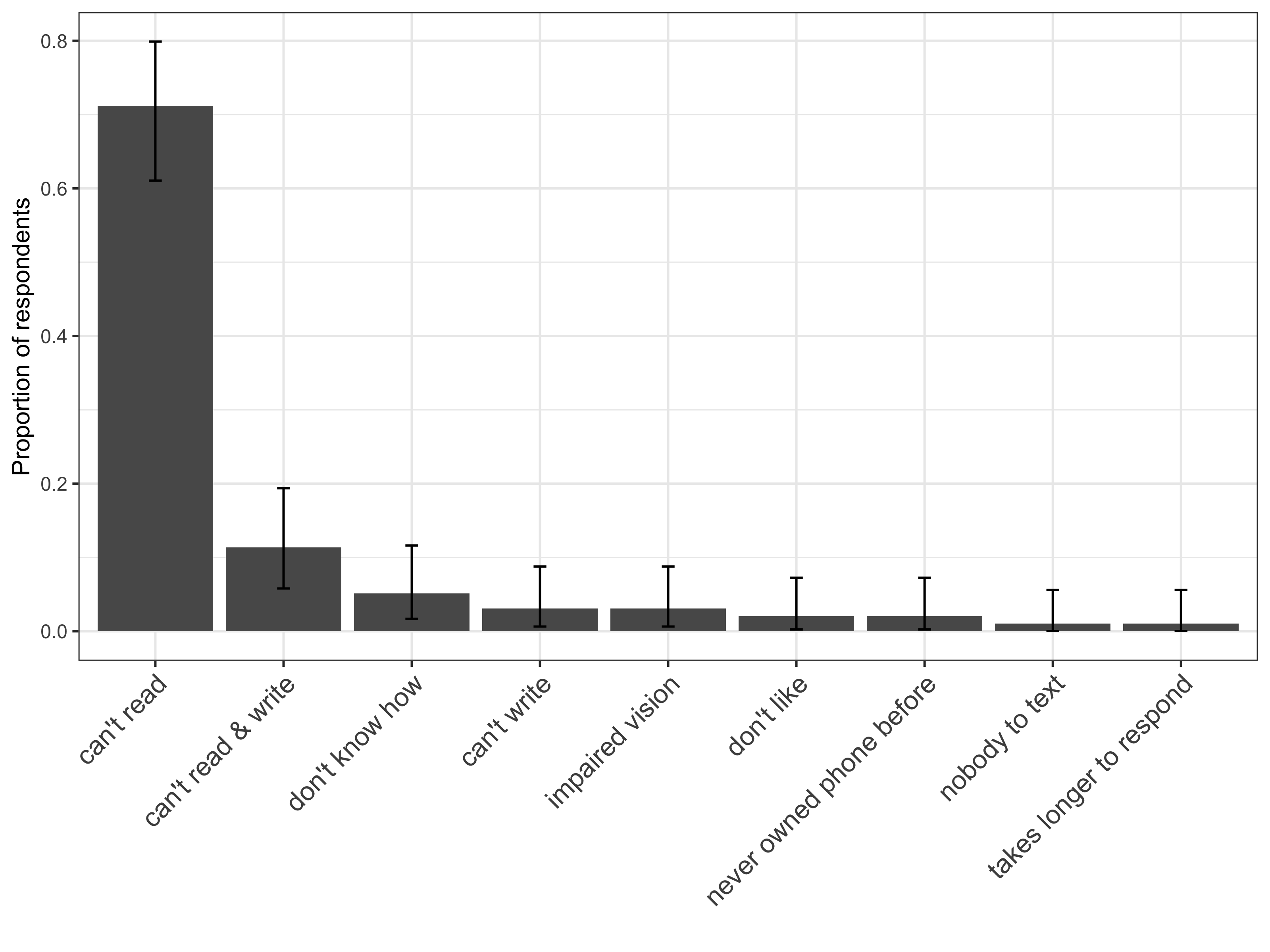

Supplement: Supplementary file 5 — Additional file 5. Figure showing reasons respondents did not use SMS. [file 40249_2020_677_MOESM5_ESM.png]

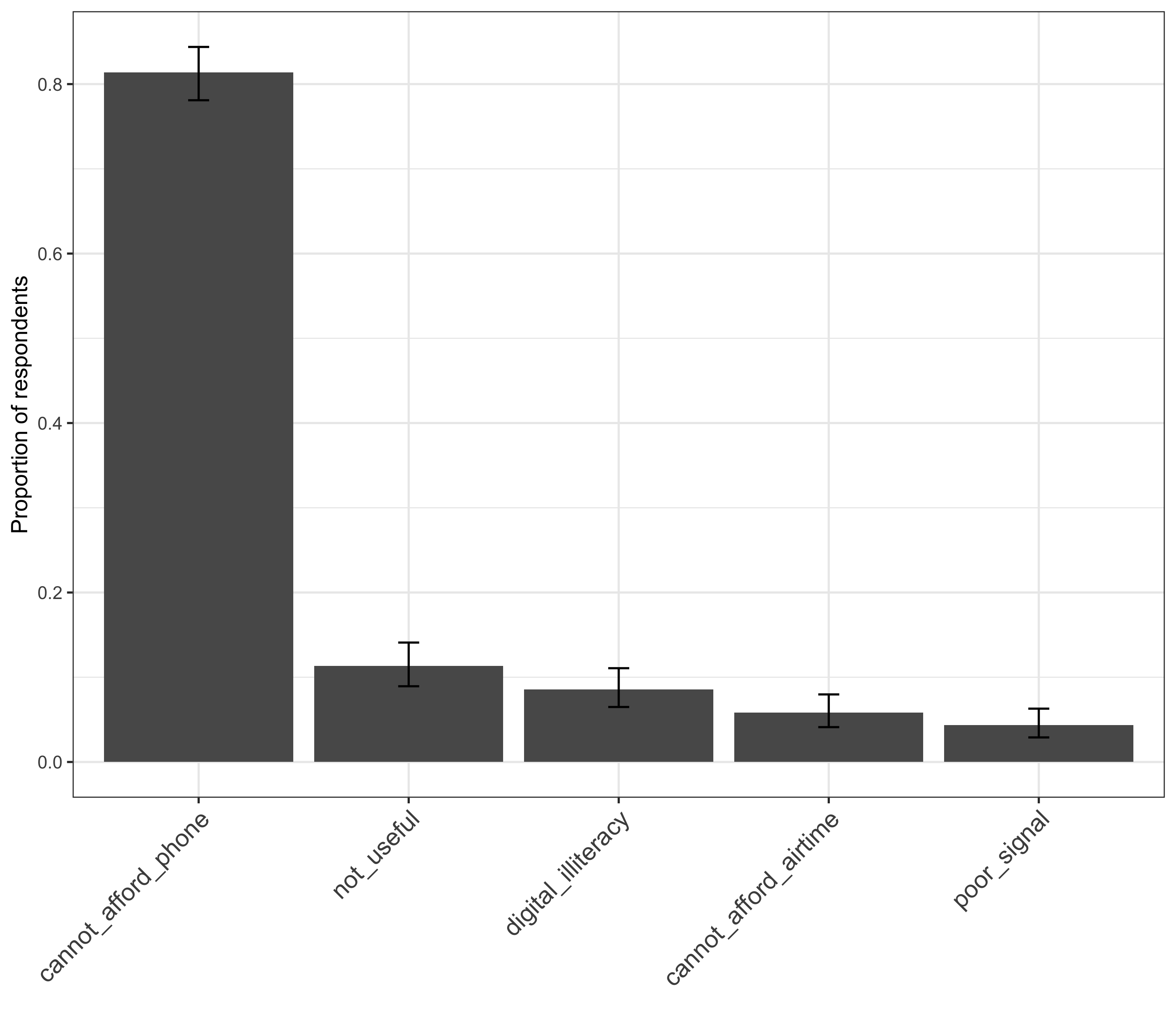

Supplement: Supplementary file 6 — Additional file 6. Figure showing reasons given for not owning a mobile phone. [file 40249_2020_677_MOESM6_ESM.png]
