# Supplementary material for: Factors associated with mobile phone ownership and potential use for rabies vaccination campaigns in southern Malawi
Source: Infect Dis Poverty. 2020 Jun 5;9:62. doi: 10.1186/s40249-020-00677-4 (PMC7275584; doi:10.1186/s40249-020-00677-4)
Supplement: Supplementary file 7 — Additional file 7. Table showing multivariable logistic regression model predicting phone ownership. [file 40249_2020_677_MOESM7_ESM.docx]

**Additional File 7: Table showing multivariable logistic regression model predicting phone ownership**

| Variable | Odds Ratio | Standard Error | 95% Confidence Interval |
| --- | --- | --- | --- |
| District: Blantyre | 1 |  | Reference |
| District: Chiradzulu | 0.53 | 0.18 | 0.37-0.75 |
| District: Zomba | 0.32 | 0.16 | 0.23-0.43 |
| Gender: Female | 1 |  | Reference |
| Gender: Male | 1.4 | 0.13 | 1.09-1.79 |
| Education: no education | 1 |  | Reference |
| Education: primary | 2.66 | 0.19 | 1.85-3.88 |
| Education: secondary | 8.44 | 0.22 | 5.48-13.18 |
| Education: college/higher | 18.69 | 0.77 | 5.1-121.01 |
| Education: no response | 0.75 | 0.49 | 0.26-1.86 |

7. Odds ratios and 95% CI estimated by multivariable logistic regression model predicting phone ownership
